# Positive-unlabeled learning identifies vaccine candidate antigens in the malaria parasite *Plasmodium falciparum*

**DOI:** 10.1038/s41540-024-00365-1

**Published:** 2024-04-27

**Authors:** Renee Ti Chou, Amed Ouattara, Matthew Adams, Andrea A. Berry, Shannon Takala-Harrison, Michael P. Cummings

**Affiliations:** 1 Center for Bioinformatics and Computational Biology, University of Maryland, College Park, College Park, MD USA; 2 Center for Vaccine Development and Global Health, University of Maryland School of Medicine, Baltimore, MD USA

**Keywords:** Immunology, Diseases

## Abstract

Malaria vaccine development is hampered by extensive antigenic variation and complex life stages of *Plasmodium* species. Vaccine development has focused on a small number of antigens, many of which were identified without utilizing systematic genome-level approaches. In this study, we implement a machine learning-based reverse vaccinology approach to predict potential new malaria vaccine candidate antigens. We assemble and analyze *P. falciparum* proteomic, structural, functional, immunological, genomic, and transcriptomic data, and use positive-unlabeled learning to predict potential antigens based on the properties of known antigens and remaining proteins. We prioritize candidate antigens based on model performance on reference antigens with different genetic diversity and quantify the protein properties that contribute most to identifying top candidates. Candidate antigens are characterized by gene essentiality, gene ontology, and gene expression in different life stages to inform future vaccine development. This approach provides a framework for identifying and prioritizing candidate vaccine antigens for a broad range of pathogens.

## Introduction

Artemisinin-based combination therapies and other tools have contributed to substantial reductions in the malaria burden in many endemic areas over the last decade^1^. However, progress toward malaria elimination has stalled as malaria incidence has plateaued and gains have been threatened by the emergence of resistance to interventions in the parasite and vector^1–4^. With the possible future exception of dracunculiasis caused by Guinea worm, no infectious disease has been completely eradicated without the aid of an efficacious vaccine^5,6^. Thus, malaria vaccines are a critical tool for malaria elimination.

*Plasmodium* parasites are transmitted to humans when infective mosquitoes take a blood meal and inject sporozoites, which develop and multiply in the liver. Vaccines directed against this pre-erythrocytic stage are meant to block infection. After emerging from the liver, *Plasmodium* merozoites invade and replicate inside red blood cells. This erythrocytic stage of the life cycle causes malaria disease and death, which blood-stage vaccines are intended to limit. Transmission-blocking vaccines would inhibit parasite sexual reproduction and development in the mosquito, preventing onward transmission^7^. Design of a broadly protective malaria vaccine has been hampered by several factors, including multiple parasite life stages that express different antigens, extensive genetic diversity within individual antigens targeted by vaccines, partial natural immunity that is short-lived and non-sterilizing, and incomplete knowledge of immune correlates of protection^8^. To date, very few malaria vaccine candidates have been evaluated in clinical trials, with most demonstrating limited efficacy^9^, including the first malaria vaccine approved for use by the World Health Organization, RTS,S, which displayed only 36% efficacy in a Phase 3 trial when given to children 5-17 months old as a primary series followed by a booster dose^10^. Another recently approved vaccine, R21, showed an efficacy of 71% in phase 1/2b^11^.

Malaria parasites are haploid in humans and briefly diploid in mosquitoes. Extensive genetic variation is generated through mutation during mitotic reproduction in humans and by sexual recombination in the mosquito. The first *P. falciparum* genome was published in 2002^12^, but nearly 20 years later most vaccine development efforts have focused on a small number of highly diverse vaccine candidates identified using traditional vaccinology approaches that identify antibody targets in immune sera, rather than a more comprehensive, genome-level approach. These highly immunogenic candidates have typically evolved extensive genetic diversity in response to immune pressure. Thus, many vaccines have displayed some degree of allele-specific efficacy (including RTS,S)^13–16^, demonstrating greater efficacy against parasites with target alleles matching those in the vaccine formulation (i.e., vaccine allele-specific efficacy)^8^.

Reverse vaccinology utilizes bioinformatics approaches to identify pathogen antigens or epitopes that could be used as vaccine candidates^17–19^. It was first proposed by Rino Rappuoli who screened the Meningococcus B proteome to identify five antigens with bactericidal activities, which were subsequently included in the licensed four-component MenB vaccine (4CMenB, Bexsero^®^)^20–23^. Reverse vaccinology has since been used to identify vaccine antigens for other bacterial and viral pathogens^24–29^. The wealth of systems data available for *P. falciparum* lends itself to the use of reverse vaccinology to identify new malaria vaccine antigens, which may allow identification of less immunodominant but more conserved antigens that have been missed using traditional vaccinology approaches based strictly on immunogenicity. There has been limited use of reverse vaccinology to identify malaria-candidate antigens. Singh et al. ^30^ applied the concept to identify candidate antigens with signal peptide and glycosylphosphatidylinositol (GPI) anchor motifs while Pritam et al. ^31^ also used signal peptide and GPI-anchor prediction tools along with T-cell epitope prediction to identify *P. falciparum* epitopes. Both studies focused on a limited number of protein or epitope properties. In contrast, machine learning in reverse vaccinology does not require a priori assumptions about the importance of specific criteria, and instead, “learns” protein properties most associated with vaccine potential based on known antigens.

Positive-unlabeled (PU) learning is applicable to many biological problems where the labeling process is often expensive or time-consuming, and only a small fraction of entities might be labeled^29,32^. Learning from the labeled positives, PU learning identifies potential positives among the unlabeled entities based on the properties of the positives^33,34^. This approach has been used to identify genes associated with human disease based on various data types, including human protein interaction data, gene expression data, gene ontology, and phenotype-gene association data^35^, yet to our knowledge, it has not been applied to identify candidate antigens. PU learning is particularly attractive for *P. falciparum*, as ~40% of genes in the genome encode proteins of unknown function^36,37^. This general vaccine candidate antigen identification framework can be applied to other pathogens, especially those having only a few known antigens identified and less characterized, such as the second-most prevalent malaria parasite species, *P. vivax*.

Here, we modify canonical positive-unlabeled random forest (PURF)^38^ to distinguish proteins with vaccine potential (i.e., antigens) from non-antigens, based on properties of known *P. falciparum* antigens, and rank the candidates with probability scores. Variable importance is assessed to understand the protein properties contributing most to identifying candidate antigens. The candidates are linked to other data types (e.g., gene essentiality^39^, stage-specific single-cell transcriptomic data^40–42^, and proximity to the known malaria vaccine antigens), to allow further characterization and prioritization in subsequent vaccine development.

## Results

### Identification of potential *P. falciparum* candidate antigens

**Fig. 1:**
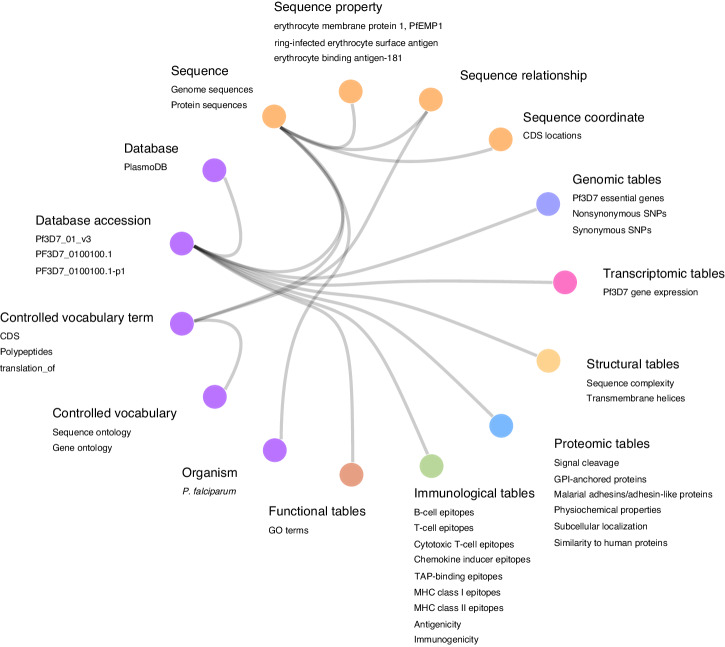
Database schema of *P. falciparum* vaccine target identification. The database is structured as a collection of data tables here represented as nodes with colors indicating different groups of tables. Part of the tables in the database are listed as examples. The lines of the hierarchical edge bundling plot show the hierarchical relationships between tables. The orders in the hierarchical structure are origin (root node), group of tables, and data table. Tables with the same type of relationship to the foreign table are collapsed into one node. Data tables generated from computational analyses are connected to sequence (purple) and basic information (orange) tables with gene accession identifiers.

In this study, 52 known antigens were selected from the intersection of the antigen sets obtained from the literature and from epitope information from the Immune Epitope Database (IEDB)^43^, based on their ability to elicit an immune response^29^. These 52 known antigens include 16 of the 19 *P. falciparum* antigens targeted by vaccine constructs that have proceeded to clinical vaccine development^44^, and four antigens–circumsporozoite protein (CSP), merozoite surface protein 5 (MSP5), 6-cysteine protein family (p230), and merozoite surface protein 5 (MSP5)–representing vaccine candidates from different parasite life stages and with varying levels of genetic diversity^45–48^ (Methods), were selected to serve as reference points for candidate antigen prioritization. A relational database was created to organize data assembled and generated for the *P. falciparum* proteins (Fig. 1; Supplementary Fig. 1). The structural, proteomic, and immunological data were generated using various bioinformatic programs (Methods and Supplementary Data 1). We also retrieved genomic, transcriptomic, and functional information from public databases such as PlasmoDB^36^. Additional variables were created by combining variables from different data types. The 272 variables comprise 28 structural variables, 121 proteomic variables, 116 immunological variables, and 7 genomic variables (Supplementary Data 1, 2).

### Training positive-unlabeled random forest models

We employed tree-based PU learning (PURF), an ensemble of individual tree models. PURF incorporates a modified impurity measure (see Methods) that estimates the probabilities of the positives and negatives based on observations in the tree node^38^. To evaluate the ensemble, we simulated fully labeled data, and estimated the receiver operating characteristic (ROC) curve, which was calculated using the probability scores (out-of-bag scores; see Methods). The estimated ROC curve was then compared with ROC curves calculated using the probability scores against the true labels and using the PU labels (Supplementary Fig. 2). The estimated ROC curves were like those of true labels (Mann–Whitney, *q* = 0.06, *n* = 5), while the ROC curves of PU labels were different from the others (*q* = 0.01 for both comparisons). This result demonstrates that even without the true label information, the ROC curve may be recovered from the score distribution.

To select the positive level (hyperparameter for prior probability of positive samples) of PURF, we trained with positive levels from 0.1 to 0.9. The positive level of 0.5 shows the highest area under the estimated ROC curve (AUROC = 0.98) (Supplementary Fig. 3). Proteins were ranked based on probability scores, which is defined as the proportion of trees in the ensemble predicting the protein to be antigenic. The overall percentile ranks (PRs) of the known antigens were highest for the ensemble with 0.1 positive level (area under the ranking curve; *AUC* = 0.83), whereas all known antigens were predicted correctly (explicit positive recall; *EPR* = 1) by the ensembles with 0.8 and 0.9 positive levels (Supplementary Fig. 4). The AUC and EPR of the ensemble with 0.5 positive level were 0.81 and 0.83, respectively.

To improve the performance, we utilized a method like the synthetic minority oversampling technique (SMOTE)^49^ to increase representation of known antigens. The weighting made known antigens equally representative by duplicating those that are more distant from others in the variable space, which increased classification performance. The estimated ROC curve showed an increase in classification separability (AUROC = 0.99, positive level = 0.5, Supplementary Fig. 5). The known antigens obtained a higher percentile rank (Supplementary Fig. 6), and the EPR of the ensemble with 0.5 positive level increased to 0.92.

### Classification tree filtering using reference antigens

**Fig. 2:**
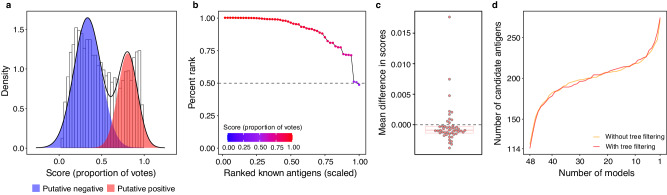
Model evaluation and validation of positive-unlabeled random forest models. **a** Score distributions of unlabeled proteins predicted by the tree-filtered model. The putative positive (red) and negative (blue) distribution groups were calculated by fitting a two-component Gaussian mixture model. A receiver operating characteristic curve (ROC) was calculated based on the putative distributions, and the area under the receiver operating characteristic curve (AUROC) was 0.99. **b** Evaluation of known antigen scores predicted by the tree-filtered model. Points represent known antigens. The *x*-axis shows the scaled ranks of the 52 known antigens. The *y*-axis notes percentile ranks (PR) of known antigens in the set containing all *P. falciparum* proteins. The dashed line indicates the 50th percentile rank. Gradient colors show probability scores. The area under the ranking curve was 0.90. **c** Distribution of mean differences in scores after known antigen label removal for the final tree-filtered ensemble. Dots represent the 48 validation iterations. The box plot shows median with first and third quartiles. The lower and upper whiskers indicate 1.5× interquartile range from the first and third quantiles, respectively. The gray dashed line conveys a zero-mean difference in scores. **d** Plot of overlapping antigens across the top 200 candidate sets generated from the validation models. The *x*-axis shows the number of validation models in reverse order, and the *y*-axis indicates the number of candidate antigens in agreement with the corresponding number of models. Line colors show data from non-tree-filtered (yellow) and tree-filtered (red) validation models, respectively.

To utilize the random forest structure to prioritize candidate antigens, we identified tree models that correctly predicted all reference antigens that were in the out-of-bag set of the tree (those proteins not used to build the tree). Trees that did not have reference antigens in the out-of-bag set or incorrectly predicted any of the out-of-bag reference positives were removed. PURF with tree filtering had an estimated AUROC of 0.99 (Fig. 2a). The evaluation of the 52 known antigens showed that 51 had percentile rank >50 and the EPR was 0.94 (Fig. 2b). For further characterization, we selected the top 200 candidate antigens with a probability score >0.94 because half of the known antigens had scores above this threshold.

To assess robustness to the inclusion of specific reference antigens, we performed an iterative validation procedure by sequentially removing the positive label from one of the 48 known antigens (excluding the four reference antigens) from each iteration as an adversarial control^50^, conducted variable space weighting, and retrained our models. The results show small mean differences in scores of the remaining known antigens before and after the label removal (Fig. 2c), and there was no significant difference between filtered and unfiltered ensembles (Mann–Whitney, *p* = 0.32, Supplementary Fig. 7). The top 200 candidate lists from the 48 ensembles were generated, and the cumulative numbers of candidates that agreed on 48, 47, 46 ensembles, and so on, are similar between the filtered and unfiltered ensembles (Fig. 2d), demonstrating that the tree filtering procedure did not affect the overall PURF structure in predicting candidate antigens.

**Fig. 3:**
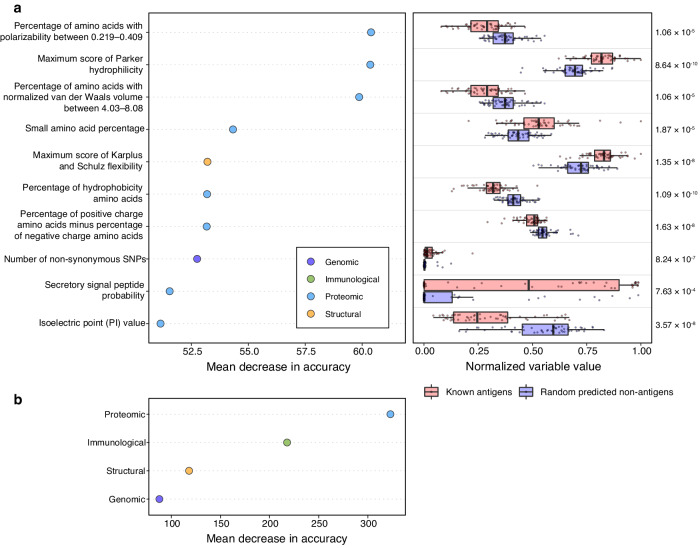
Positive-unlabeled random forest model interpretation based on known antigens. **a** The left panel displays permutation-based variable importance analysis of the final tree-filtered model. The *x*-axis shows the mean decrease in accuracy (scaled by the standard error) of the known antigen set (*n* = 52) after permuting the variables for each tree in the model. The *y*-axis lists the ten most important variables in predicting the known antigens. The property groups of the variables are noted by colors. The right panel shows a summary of variable values of the known antigens (red) and randomly selected proteins (*n* = 52; blue) that are predicted as non-antigens by the final tree-filtered model. The ten most important variables obtained from the permutation-based variable importance analysis are shown. Points represent proteins. Boxplots show median with first and third quartiles, and the whiskers indicate the 1.5 interquartile range extended from the first and third quartiles. Numbers on the right show adjusted *p*-values calculated using two-sided Mann–Whitney tests. Variable values were normalized based on the entire data set. **b** Permutation-based group variable importance analysis. Variable importance was calculated on the known antigens, and the decrease in accuracy after variable permutation was recorded. Variables in the same property groups were permutated together. The mean decrease in accuracy was standardized using the standard error computed across all trees in the model.

To understand protein variables contributing to the identification of the known antigens, we investigated the mean decrease in prediction accuracy across all trees in the filtered ensemble with variable permutations. All variables were used to identify antigen candidates, and the top ten most important variables include one structural, one genomic and eight proteomic variables (Fig. 3a). Comparisons of the variable values between the known antigens and 52 random proteins predicted to be non-antigens by tree-filtered PURF reveal that the known antigens contain fewer amino acids with high polarizability (K, M, H, F, R, Y, W), comprise fewer amino acids with high van der Waals volume (M, H, K, F, R, Y, W), and have fewer hydrophobic amino acids (C, L, V, I, M, F, W) (Fig. 3b). Moreover, the known antigens have fewer positively charged amino acids (K, R) and a lower isoelectric point value (Fig. 3b). Known antigens also have a higher secretory signal peptide probability, a higher number of non-synonymous SNPs, and have higher flexibility and hydrophilicity for predicted epitopes (Fig. 3b). The importance of variables grouped by data categories showed that the proteomic variables are most important in identifying known antigens (Fig. 3c).

### Proximity of top-ranked candidates to reference antigens

**Fig. 4:**
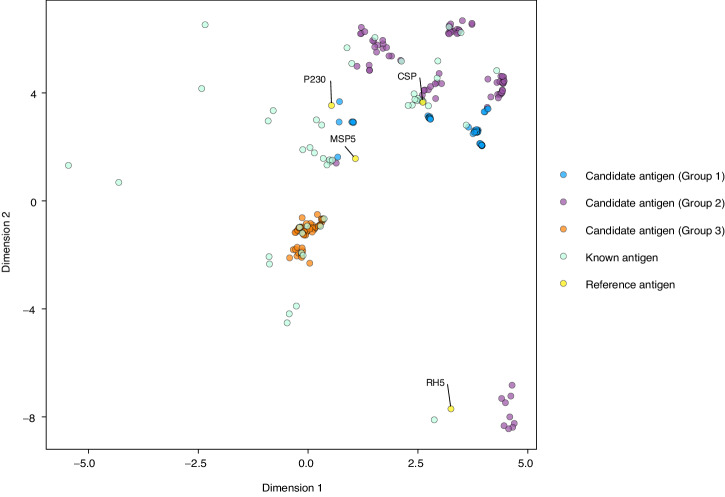
Clustering of top 200 candidate antigens based on proximity measured from tree-based model. First two dimensions of UMAP are shown. Top 200 candidate antigens from the final tree-filtered model were grouped based on *k*-means clustering. Points represent top 200 candidate antigens in three groups, 48 known antigens (light cyan), and four reference antigens (yellow; protein names noted by text).

To understand how tree filtering assisted in prioritizing antigen candidates based on the reference antigens, we examined the proximity space before and after tree filtering. Proximity values are the proportion of times a pair of proteins occur in the same terminal node of a tree model and represent the similarity with respect to variables used in the model. The proximity was converted to an Euclidean distance (smaller values indicate more closeness) and visualized using multidimensional scaling. The top candidate antigens were clustered into three groups (Fig. 4). The probability scores of candidate antigens in groups 1 and 2 increased after tree filtering (Supplementary Fig. 8), indicating that some candidates in groups 1 and 2 have been prioritized into the top candidate list after tree filtering.

To study the relationships between the candidate and reference antigens, we compared the Euclidean distances of the candidate antigens in each group to each of the four reference antigens. The distances significantly changed (FDR < 0.05) in all three groups after tree filtering. Comparing the three groups, after tree filtering, group 3 had the farthest median distance to the reference antigens, group 1 had the closest median distance to CSP, MSP5, and P230, group 2 is closest to RH5 (red points in Supplementary Fig. 9). For RH5, MSP5, and P230, both groups 1 and 2 moved closer to the three reference antigens (blue and purple points in Supplementary Fig. 9) and group 3 moved further away after tree filtering (dark orange points in Supplementary Fig. 9), suggesting that reference antigens may have less effect on prioritizing group 3 antigen candidates. Overall, RH5, MSP5, and P230 may have positive influences on the prioritization of group 1 and group 2 antigen candidates. Interestingly, the median distances of group 2 antigen candidates are less than 0.5 to all reference antigens (red points in Supplementary Fig. 9), suggesting that over half of the trees in PURF agreed on the protein similarities between group 2 and all four reference antigens.

### Variable importance of candidate antigen groups

Permutation-based variable importance analyses were conducted for each of the three candidate antigen groups. The shared importance variables in identifying the candidates as antigens for the three groups includea higher number of non-synonymous SNPs, higher flexibility and hydrophilicity for predicted epitopes, lower probability of mitochondrial subcellular localization, and a smaller number of hydrophobic amino acids (Supplementary Fig. 10). The shared important properties of candidate antigens in the three groups were similar to the properties of known antigens. Among the three groups, group 2 had the most similar important variables as known antigens. The secretory signal peptide probability, which was ranked ninth in the important variable list for known antigens, was ranked 83rd, 226th, and 83rd in the results of groups 1, 2, and 3, respectively, suggesting that a secretory signal peptide may be important in classifying proteins as antigens (probability score $\ge$0.5), but is not as critical for a higher probability score ($\ge$0.9).

In terms of top-ranked variables, the number of non-synonymous SNPs, epitope flexibility, and epitope hydrophilicity were ranked among the top three for groups 1 and 2, and among the top four for group 3 (Supplementary Tables 1–3). The median number of non-synonymous SNPs is lower and with a smaller variance in the distribution for group 2 compared to groups 1 and 3 (Supplementary Fig. 10). The predicted number of B-cell epitopes in outer membrane regions was ranked as the most important variable for group 3, whereas it was the least important among the 272 variables for groups 1 and 2 (Supplementary Table 3).

### Characteristics of identified potential vaccine antigen targets

We applied gene ontology (GO) enrichment analysis to assess annotation-associated properties of candidate antigen groups compared to the background of the *P. falciparum* proteome. Group 1 was significantly enriched for genes encoding proteins involved in cell-cell adhesion, cytoadherence to the microvasculature, erythrocyte aggregation, and antigenic variation. Similar enriched GO terms were observed for group 3 (Table 1). Group 2 candidate antigens were enriched in the parasite nucleus and cytoplasm and not associated with antigenic variation (Table 1), suggesting these potential antigens may be less immunogenic or less exposed to the host immune response. Further examination of the gene products of group 1 revealed that 85% of the candidates are erythrocyte membrane proteins (PfEMP1, Supplementary Data 3), whereas 36% and 26% of candidates in groups 2 and 3, respectively, are conserved proteins with unknown functions (Supplementary Data 3).

**Table 1:** Significantly enriched gene ontology terms with false discovery rate (FDR) <0.05 in gene ontology enrichment analysis of candidate antigen groups with the background proteome of *P. falciparum* 3D7

		GO term	Number of genes	−Log_10_FDR
Group 1 (61 candidates)	Biological process	Cell-cell adhesion	45	4.12
		Cytoadherence to microvasculature, mediated by symbiont protein	43	3.55
		Modulation by symbiont of host erythrocyte aggregation	42	3.53
		Antigenic variation	43	3.50
	Cellular component	Host cell plasma membrane	44	4.74
		Infected host cell surface knob	44	4.74
		Integral component of membrane	54	3.91
		Maurer’s cleft	5	1.34
	Molecular function	Cell adhesion molecule binding	44	4.45
		Host cell surface receptor binding	51	3.71
		Protein binding	8	2.65
Group 2 (83 candidates)	Biological process	Chromatin remodeling	4	3.95
		Regulation of transcription, DNA-templated	7	3.19
		Positive regulation of transcription, DNA-templated	2	1.53
	Cellular component	Nucleus	45	3.57
		Cytoplasm	18	3.51
		Membrane	9	2.87
		Extracellular region	3	2.08
		Chromosome	2	1.44
		Rhoptry neck	2	1.44
		P-body	2	1.34
		Vesicle	2	1.34
	Molecular function	DNA-binding transcription factor activity	7	4.65
		ATP binding	12	4.06
		DNA binding	9	4.06
		Sequence-specific DNA binding	6	4.06
		Protein binding	21	3.90
		Actin binding	3	2.52
		Chromatin binding	3	2.19
		Protein phosphatase regulator activity	2	2.01
		Histone-lysine N-methyltransferase activity	2	1.69
		Calcium ion binding	3	1.67
Group 3 (56 candidates)	Biological process	Cell-cell adhesion	6	4.35
		Entry into host	5	3.54
		Protein phosphorylation	4	2.09
		Response to xenobiotic stimulus	4	1.81
		Cytoadherence to microvasculature, mediated by symbiont protein	4	1.46
		Modulation by symbiont of host erythrocyte aggregation	4	1.41
		Cell motility	2	1.37
		Antigenic variation	4	1.37
	Cellular component	Integral component of membrane	47	4.26
		Nucleus	13	4.26
		Membrane	16	4.23
		Infected host cell surface knob	4	3.56
		Host cell plasma membrane	5	2.72
		Apicoplast	5	1.79
		Rhoptry neck	2	1.79
		P-body	2	1.66
		Cytoplasm	7	1.43
	Molecular function	Heparin binding	4	3.84
		Host cell surface receptor binding	7	3.84
		Cell adhesion molecule binding	4	3.29
		Protein kinase activity	4	2.36

We filtered the candidate antigen groups by gene essentiality, where genes with mutagenesis index score < 0.5 were retained^39^. We examined the expression of the genes encoding the remaining candidate antigens in different *P. falciparum* life stages based on single-cell transcriptomic data from the Malaria Cell Atlas^40–42^. Of the group 1 candidates remaining after essentiality filtering, one was expressed mainly in the blood stage, and the other was expressed in all life stages, with higher expression levels in a larger portion of cell populations in the blood and gametocyte stages (Supplementary Fig. 11). For groups 2 and 3, most candidate antigen genes were expressed primarily in the blood, gametocyte, and ookinete stages, and a smaller number of groups 2 and 3 candidates were expressed in all life stages (Supplementary Fig. 11).

## Discussion

Over the past decades, various malaria vaccine candidates have been developed and proceeded to clinical trials. Nevertheless, a highly efficacious and long-lasting malaria vaccine against *P. falciparum* is still an unmet need. We are now in the second wave of malaria vaccine development^51^, with the goal of selecting vaccine antigens with potential to elicit an enhanced immune memory response and a protective efficacy of at least 75% against clinical malaria^52^. With the advancement of genome sequencing of *Plasmodium* and bioinformatics tools, reverse vaccinology has become a viable vaccine development approach for this complex organism.

Reverse vaccinology has been applied using sequential filtration of protein properties or with machine learning, both of which have identified potential new vaccine antigens for *Plasmodium* species, but with some limitations^31,53^. Approaches based on sequential filtration lack standardized filtering criteria, with thresholds often selected based on empirical evidence, and could be difficult to generalize when there are many protein variables^29,54^. In *P. falciparum*, there are only a small number of known antigens that can be labeled as positives, and non-antigens are difficult to identify from the literature or based on reference genomes with incomplete annotation. One study using machine learning algorithms to predict potential vaccine antigens in eukaryotic pathogens only examined seven protein variables and did not consider genome properties such as sequence complexity and genetic diversity^53^, both of which are relevant to malaria vaccine development and have impacted the efficacy of first-generation malaria vaccines^13–16^. Additionally, this study examined only a relatively small set of three *Plasmodium* proteomes (73 antigens and 51 non-antigens, from *P. falciparum*, *P. yoelii yoelii*, and *P. berghei*). In contrast, we performed comprehensive analyses on 5393 *P. falciparum* proteins and computed 272 protein variables on each. To ensure a high-quality PU data set of known *P. falciparum* antigens, we took the intersection of antigen sets curated from the literature and IEDB^43^.

PU learning takes advantage of unlabeled data and improves modeling when only a small portion of entities are labeled as positive^33,55^. In this study, we chose random forest^56^ as the basis for our PU learning because of its high predictive accuracy, high interpretability, and insensitivity to outliers and predictive variable scales^57^. Additionally, PURF is amenable to the modifications we developed here. Permutation-based variable importance analysis is naturally derived from the random forest architecture and imparts a quantitative measure of the variable importance^56^. Moreover, many studies involving machine learning analyses focus primarily on the model accuracy and develop complex models that are hard to interpret. However, it is critical to understand the relationships learned by the model and whether they are biologically meaningful^50,58^. Here, the interpretation of PURF provides helpful insights on how the models have learned in distinguishing known antigens from non-antigens, and how the previously unknown candidate antigens were identified. Although PU learning enabled us to fully harness the entire *P. falciparum* proteome, it is a data-driven approach that could be affected by the known antigens provided. Thus, in this study, efforts were made to ensure the quality of the known antigens. Further inclusion of more high-quality known antigens may improve the model performance.

The approach described in this study identified previously unknown vaccine candidate antigens for *P. falciparum* vaccine development. The research scheme provides a flexible framework, in which the candidate antigens can also be prioritized using a different set of reference antigens selected using other criteria, while not affecting the overall PURF structure. Candidate antigens identified in this study have been filtered based on gene essentiality, where mutations in these genes could affect parasite viability, and thus may help reduce parasite escape from vaccine-induced immune responses^39^. Most candidate antigens were expressed predominantly in a single life stage, which is consistent with the observations of previous studies^59^. For instance, group 3 antigens were mostly expressed in blood and sexual stages, which were associated with a larger number of B-cell epitopes in the outer membrane regions. However, some candidates were expressed in multiple life stages, which may make them attractive vaccine antigens because they would target multiple life stages. An interactive summary report of the candidate antigens identified is available online (https://mrp-bioinformatics.github.io/malaria_antigen_candidates/ and in Supplementary Data 3). The information about the closest known antigens to the candidates and single-cell gene expression is also included. For future studies, further filtering criteria, such as isoelectric point, molecular weight, and folding propensity, may be applied to select candidate antigens for heterologous protein expression in other species systems to perform functional assays^60,61^.

Our approach exploits PU learning in reverse vaccinology to identify potential *P. falciparum* vaccine candidate antigens for future vaccine development, which does not assume filtering criteria of protein variables, is driven by the proteome, and leverages a small set of known antigens. The alteration of the model ensemble based on the reference antigens aids in candidate antigen prioritization. In response to the shift in species constitution in malaria-endemic areas, the developed framework can be expanded to *P. vivax* and other *Plasmodium* species that cause human malaria^62^. The methodology can be further tailored and applied to other disease pathogens. More broadly, beyond vaccine development, the study may also inspire other scientific research areas, if there is only a relatively small amount of evidence collected to guide the prioritization of the study entities.

## Methods

### Known antigen protein collection

Known antigens were selected based on literature and epitope information. Covidence (www.covidence.org), a web-based application tool designed for systematic review and streamlined screening of the literature, was used to select, and extract literature covering malaria vaccine research. Our goal was to look for all the malaria vaccine candidates that have been already reported in the literature. The search terms include the following: “malaria vaccine”, “malaria vaccine candidate”, “malaria vaccine antigen”, “malaria vaccine protein”. In brief, the search covered papers and documents having both malaria and vaccine in any of its sections. The search generated a set of articles that discuss malaria vaccine candidates, rather than a list of each of the candidates. Overall, our search produced 7415 articles in total. We then manually examined these papers to identify proteins used as malaria vaccine candidates. Non-redundant candidates were selected based on gene names, GenBank ID, or aliases.

The known antigens selected based on the epitope information were extracted from the PlasmoDB^36^ immunology section. Epitopes from the Immune Epitope Database (IEDB)^43^ are mapped to the PlasmoDB proteins with exact string matching; at the same time, the corresponding GenBank proteins from IEDB were aligned to PlasmoDB proteins using BLAST^63^. The similarity threshold of a best hit is percent identity ≥97%. We selected proteins from PlasmoDB as known antigens if the protein has a similarity score larger than or equal to the similarity threshold, or having all listed epitopes aligned exactly to the PlasmoDB protein sequence. The set based on the literature contained 177 known antigens, and the set based on the epitope information had 373 known antigens. The final known antigen list was an intersection of the two sets and included 52 antigen proteins.

The set of 52 known antigens includes 16 of the 19 *P. falciparum* antigens targeted by vaccine constructs that have proceeded to clinical vaccine development^44^. Based on the criteria above, Pf11-1 (PF3D7_1038400), LSAP2 (PF3D7_0202100), and VAR2CSA (PF3D7_1200600) were not among the known antigens; however, VAR2CSA was among the top 200 predicted antigens resulting from the analysis). Four of the known antigens were selected as reference antigens to help better understand our models. Circumsporozoite protein (CSP) is a surface protein expressed during the pre-erythrocytic stage and is the active component of the WHO-approved RTS,S and R21 vaccines^64,65^. Reticulocyte binding homolog 5 (RH5) is expressed in the blood stage, functions as an invasion ligand, and is currently under malaria vaccine development^66,67^. Merozoite surface protein 5 (MSP5) is expressed in sporozoites, late liver stages, and in blood stage parasites, and has recently been shown to elicit antibodies strongly associated with protection following vaccination with PfSPZ sporozoite vaccines^68^. P230, in the 6-cysteine protein family, is expressed and located on the surface of gametocytes^69^. It is a leading transmission-blocking vaccine candidate shown to induce serum functional activity in humans^70^, including in a recent Phase 1 trial in Mali^71^. These reference antigens display a range of genetic diversity, as measured by percentile rank of SNPs per Kb coding sequence over *P. falciparum* proteome (P230 0.39, MSP5 0.43, RH5 0.52, CSP 0.94).

### Collection of *Plasmodium* data and bioinformatic analyses

*P. falciparum* 3D7 genome information and protein sequences were collected from PlasmoDB^36^ release 43 (2019-04-25). An in-house database was constructed using MariaDB version 10.3.22 (https://mariadb.com/). The data tables are connected via table identifiers or gene accessions. Part of the Chado schema from the Generic Model Organism Database^72^ was integrated into the database design to eliminate redundancies. The database contains eight categories of tables, including basic information, sequence information, genomic, transcriptomic, functional, structural, proteomic, and immunological tables. See Supplementary Data 1 for detailed information on variable name, data type, program or tool, description, collection method, collection date, and source.

In brief, the reference genome, coding sequences (CDS), and protein sequences were directly downloaded from PlasmoDB^36^. Proteins with stop codons within the sequence or derived from pseudo genes were removed. Protein sequences having “X” symbols were also removed. Selenocysteines in selenoproteins were replaced with cysteines for downstream bioinformatic analyses. The preprocessing resulted in 5393 *P. falciparum* proteins. General information including genome, coding sequence locations, protein sequences, and sequence ontology terms were stored in the basic information and sequence information database tables.

For genomic data tables, single nucleotide polymorphisms (SNPs) discovered from next-generation sequencing were directly downloaded from PlasmoDB^36^ under the genetic variation section (365 genomes collected; 2 Central Africa, 3 Central America, 12 East Africa, 1 Northeast Africa, 174 South America, 1 Southeast Africa, 7 Southeast Asia, 163 West Africa, 1 Western Europe, and 1 of unknown geographic origin). The measures of SNPs include the total number of SNPs, number of non-synonymous SNPs, number of synonymous SNPs, number of nonsense SNPs, number of non-coding SNPs, ratio of non-synonymous to synonymous SNPs, and number of SNPs per kb coding sequence. Gene essentiality measured from saturation-level mutagenesis was obtained from the literature^39^. Transcriptomic data included DNA microarray^73^ and bulk RNA-seq^74–77^ data at various *P. falciparum* life stages retrieved via PlasmoDB, and single-cell RNA-seq data from the Malaria Cell Atlas^40–42^. Functional data including gene ontology terms were downloaded directly from PlasmoDB^36^ as a GAF file.

For structural data, transmembrane helices were predicted using the TMHMM version 2.0 web server (http://www.cbs.dtu.dk/services/TMHMM/)^78,79^. Sequence complexity was analyzed using the SEG^80^. Beta-turns, surface accessibility, and flexibility were analyzed using IEDB Antibody Epitope Prediction version 3.0^81–83^. By combining the results from TMHMM and SEG, new protein variables of sequence complexity in the outer-membrane (non-cytoplasmic), transmembrane, and inner-membrane (cytoplasmic) regions were generated.

For proteomic data, subcellular localizations were predicted using the CELLO version 2.5 web server (http://cello.life.nctu.edu.tw)^84^. Malarial adhesins/adhesin-like proteins were predicted using the MAAP web server (http://maap.igib.res.in/index.php)^85^. Physicochemical properties were analyzed using the R packages *Peptides* version 2.4.1^86^ and *protr* version 1.6.2^87^, and IEDB Antibody Epitope Prediction version 3.0^88^. Glycosylphosphatidylinositol (GPI)-anchored proteins were predicted using the PredGPI web server (http://gpcr.biocomp.unibo.it/predgpi/pred.htm)^89^. Protein signal cleavage prediction was analyzed using the SignalP version 5.0 web server (http://www.cbs.dtu.dk/services/SignalP/index.php)^90^. Protein solubility information was obtained using the protein-sol abpred^91^. N- and O-linked glycosylation sites were predicted using GlycoEP^92^. The results of glycosylation sites were combined with the transmembrane predictions to generate additional variables of glycosylation sites in the outer-membrane, transmembrane, and inner-membrane regions. Similarity to human proteins was analyzed using BLASTP version 2.8.1+^93^.

For immunological data, T cell epitopes were predicted using the PREDIVAC web server (http://predivac.biosci.uq.edu.au/cgi-bin/population.py)^94^, which predicted epitopes specifically for sets of HLA class II allelic variants from ten population regions. B cell epitopes were analyzed using BepiPred version 2.0, BepiPred version 1.0, and ABCpred^95–97^. Additional variables of B cell epitopes in the outer-membrane, transmembrane, and inner-membrane regions were computed using the transmembrane information from TMHMM. Cytotoxic T cell epitopes were analyzed using CTLPred^98^. Chemokine inducer epitopes were analyzed using IL-10Pred^99^. Transporter associated with antigen processing (TAP)-binding peptides were predicted using TAPPred^100^. MHC class I and class II epitopes were predicted using IEDB MHC-I Binding Predictions version 2.22.3^101^ and IEDB MHC-II Binding Predictions version 2.22.3^102^, respectively. Epitope antigenicity was analyzed using IEDB Antibody Epitope Prediction version 3.0^103^, and epitope immunogenicity was predicted using IEDB Class I Immunogenicity version 1.1^104^. In general, the epitope information was summarized for each protein with the total number of epitopes passed the default threshold, and the maximum, mean, and minimum scores of the epitopes.

### Data set assembly

The data set contains the predictor variables, and the response variable labels. The variables were assembled by retrieval from the database. Antigen labeling information was added as the response variable, where proteins selected as known antigens were labeled as positive and the other proteins were unlabeled. The number of proteins was 5393, and the number of known antigens as labeled positives was 52. In total, 272 predictor variables were retrieved from the database (Supplementary Data 2). All predictor variables are of numeric type, and missing values in the variables were imputed by replacement with variable medians.

### Positive-unlabeled simulation

The simulated data were generated using the function *make_classification* from the Python *scikit-learn* package^105^. The number of proteins was 5000 and the number of predictor variables was 300, comprising 250 informative variables, 40 redundant variables, and 10 repeated variables. The response variable contained two classes (positive and negative) and was treated as true labels. Because the *P. falciparum* data set had 52 labeled positives (known antigens) out of 5393 proteins, the data set was 99% unlabeled. To convert true labels to positive-unlabeled (PU) labels, a regular random forest classifier with 1000 trees was trained to obtain probability (out-of-bag) scores for all proteins. We then randomly selected 50 proteins that were predicted to be positive by the regular random forest. We retained the positive labels of these 50 proteins and made the remaining 4950 proteins unlabeled.

### Positive-unlabeled random forest algorithm implementation

The positive-unlabeled random forest (PURF) framework is based on a modified splitting criterion called positive-unlabeled Gini index (PUGini)^38^, which is derived from the Gini criterion (Gini = 1 –∑_*j*_ ${p}_{j}^{2}$, where *p*_*j*_ is the probability of being classified as a class *j*)^106^. The new splitting criterion estimated probabilities of positive and negative proteins according to the numbers of labeled positives and unlabeled proteins in the tree node. The probabilities of positive (*p*_1_) and negative (*p*_0_) proteins were respectively estimated by the following equations^38,107^, *p*_1_ = min(|POSnode| × PosLevel × |UNL | , 1) | POS| |UNLnode | , and *p*_0_ = 1 − *p*_1_, where |POSnode| and |UNLnode| are, respectively, the numbers of labeled positives and unlabeled proteins in the node, and |POS| and |UNL| are, respectively, the numbers of labeled positives and unlabeled proteins in the data. Because PURF is based on random forest^56^, it inherits the properties of robustness to outliers and variable errors, insensitivity to monotonic transformation of variables, and high predictive power. In this study, we implemented the PURF algorithm by extending the ensemble and tree modules in the Python scikit-learn package^105^ and developed a lightweight Python package. The framework proposed by Li and Hua^38^ was slightly modified where the positive level (PosLevel) has become a hyperparameter that can be explicitly tuned by the user. We also added class functions that take tree weights as an argument to calculate probability scores with the tree filtering procedure. For the initial modeling, positive levels were set to 0.1, 0.2, 0.3, 0.4, 0.5, 0.6, 0.7, 0.8, and 0.9. The forest size was 100,000 trees.

### Positive-unlabeled random forest evaluation

Because in a PU learning problem, we do not know the true state for the unlabeled proteins, we cannot calculate the traditional evaluation metrics such as those involving true negative and false positive rates. Further, the metrics based on PU labels could be affected by the proportion of labeled positives^108^. Thus, in this study we used the following two criteria, which utilize the probability score distribution from PURF and the percentile rank of labeled positives, respectively, to examine the model performance.

The first criterion involves estimating the putative true and false positive rates from the probability score distribution to calculate the receiver operating characteristic (ROC) curve. As the probability score distribution of unlabeled proteins is bimodal, the distribution can be described using a two-component mixture with the formula: *h*(*x*) = π*h*_1_(*x*) + (1 − π)*h*_0_(*x*), where π ∈ (0,1) and x ∈ *X*, *X* being the set of all possible proteins, *h*_*1*_ is the score distribution of putative positive proteins, and *h*_*0*_ is the score distribution of putative negative proteins. In this study, the two-component Gaussian mixture was computed using the R package *mixR*, version 0.2.0^109^, and the area under the receiver operating characteristic curve (AUROC) was calculated using the R package *pracma*, version 2.3.8^110^.

The second criterion calculates the percentile rank of labeled positives (known antigens) among all protein proteins based on the probability scores. The criterion also reports the proportion of labeled positives that are correctly predicted (explicit positive recall; EPR)^55,111^. The area under the percentile rank curve was computed using the R package *pracma*, version 2.3.8^110^.

### Variable space weighting

Because only ~1% of the data were labeled as positive, the scarcity of the labeled positive may not well represent the positive (antigen) population. To make all known antigens equally representative for learning the antigen properties, a variable space weighting procedure was performed before training. A vector of variable medians was generated according to the variable set of the known antigens. The vector represents the center point^112^, which is a generalized geometric median in higher-dimensional data, of the known antigens in the variable space. The Euclidean distance between each known antigen and the center point was then calculated. The distances were scaled to rounded integer values from 1 to 10. A new data set was generated by duplicating the known antigens with the transformed distances. The number of labeled positives after variable space weighting was 122.

### Ensemble constituent filtering

The tree filtering was conducted using four selected reference antigens (CSP, MSP5, P230, RH5) to prioritize top-scored unlabeled proteins. To select trees that correctly predicted the reference antigens in the out-of-bag set, trees having no references, or which incorrectly predicted any of the out-of-bag antigens were removed, resulting in 74,089 trees filtered from the original 100,000 trees. The probability scores were recalculated using the function _set_oob_score_with_weights, where the removed trees were assigned with a weight of zero. The resulting probability scores are available in Supplementary Data 4.

### Positive-unlabeled random forest validation

Known antigens, excluding the four reference antigens, were converted to unlabeled proteins iteratively. For each of the 48 iterations, a variable-space-weighted data set was generated, and an ensemble with a positive level of 0.5 determined through hyperparameter tuning and 100,000 trees was trained. The model was subsequently processed using the ensemble constituent filtering procedure. The probability scores of the remaining known antigen predicted by both unfiltered and filtered models were recorded. The differences in scores compared to the ensembles with no antigen label removal were calculated, and the mean of these differences were then computed for each iteration. Finally, the mean differences in scores from unfiltered and filtered models were compared using a two-sided pairwise Mann–Whitney test. Additionally, the top 200 unlabeled proteins ranked based on probability scores were selected for each validation model, and the number of proteins identified in *n*, $n-1$, $n-2$, … rank lists were reported (*n* = 48). The validation results can be found in Supplementary Data 5, 6 and Supplementary Fig. 7.

### Candidate antigen clustering and comparisons

To calculate the proximity matrix^56^ for the final tree-filtered forest with 74,089 trees, a matrix was computed using the Python function apply. The matrix is symmetric with rows and columns corresponding to proteins, and a cell value of 1 indicating that the paired proteins end up in the same terminal nodes of a tree. The proximity matrix was then computed by dividing the number of trees for which the paired proteins were in the out-of-bag set. The proximity matrix was converted to a Euclidean distance matrix by subtracting the proximity value from 1. The distance matrix was further converted to a (5393 – 1)-dimensional space using multi-dimensional scaling (MDS) with the R function cmdscale. The variance explained for each dimension was calculated by dividing the eigenvalue by the sum of all positive eigenvalues.

The top 200 candidate antigens were selected from the final ensemble. A *k*-means clustering analysis was performed on the subset of the multi-dimensional data set containing the top 200 candidate antigens. To select the optimal number of clustering groups, the Gap statistic^113^ with the Tibshirani criterion^114^, Silhouettes^115^, and Elbow (or total within sum of square) methods were used. The number of clusters selected by the three methods were 3, 2, and 3, respectively. Thus, the top 200 candidates were clustered into three groups, and visualized along with the known antigens and reference antigens on the first two dimensions of the uniform manifold approximation and projection (UMAP)^116^ matrix.

For the three candidate antigen groups, we quantified three measures comparing candidate antigens between non-tree-filtered and tree-filtered ensembles: 1) probability scores; 2) Euclidean distances from the candidate antigens to each of the four reference antigens; and 3) differences in distances. For these comparisons, we used multiple pairwise Mann–Whitney tests (probability scores and Euclidean distances), and Mann–Whitney test (differences in distances), with *p* values adjusted by the Benjamini–Hochberg method^117^.

### Variable importance analyses

Permutation-based variable importance^56^ was calculated for the 52 known antigens, 61 group 1 antigen candidates, 83 group 2 antigen candidates, and 56 group 3 antigen candidates. For each tree in the forest, the prediction accuracy was recorded for the out-of-bag target proteins (e.g., the 52 known antigens). For each of the 272 variables, the variable values were permuted for all 5393 proteins, the tree was then used to predict the response of the permuted data set, and the prediction accuracy for the out-of-bag target proteins was calculated. The difference in prediction accuracy before and after variable permutation was recorded for each variable permutation. After iterating through all trees in the forest, the results from each tree were weighted according to ensemble constituent filtering (filtered trees have a weight of zero), and the weighted average decrease in accuracy and the corresponding standard error were calculated for each variable across all trees. The final mean decrease in accuracy was scaled by dividing the values by the standard error. For the importance analysis for variables grouped by data categories, the variables were grouped based on data properties (genomic, structural, proteomic, and immunological). When calculating the importance of each data category, the grouped variables were permuted together, and the decrease in prediction accuracy was measured after permutation.

### Variable value comparisons of top important variables

To compare variable values, a set of non-antigens predicted by the final tree-filtered ensemble with the same size as the target proteins (known antigens or candidate group antigens) were randomly selected. The variable values of the target proteins and randomly selected non-antigens were compared using a two-tailed Mann–Whitney test for all 272 variables. The *p*-values were adjusted for multiple tests using the Benjamini–Hochberg procedure^117^. The variable values were normalized to be between 0 and 1 based on the original data set with 5393 proteins for better visualization. The top ten most important variables based on the permutation-based variable importance analysis were visualized.

### Gene ontology enrichment analysis

Candidate antigen groups were analyzed separately using the function GOEnrichmentStudyNS in the *GOATOOLS* Python package^118^. The GAF files containing associated gene ontology terms of *P. falciparum* 3D7 genes were retrieved from PlasmoDB^36^ release 59 (2022-08-30). The directed acyclic graph file of gene ontology was downloaded from the Gene Ontology website (http://geneontology.org/docs/download-ontology/)^119,120^. The argument propagate_counts was set to false for more conservative results. The *p*-values generated from multiple Fisher’s exact tests were adjusted using the Benjamini–Hochberg method (or false discovery rate; FDR)^117^. The significance cut-off was set at 0.05.

### Candidate antigen characterization

Candidate antigens in each of the three groups were further filtered based on gene essentiality that measured from saturation-level mutagenesis of *P. falciparum*; the threshold of MIS < 0.5 was chosen as described in the original paper^39^. After filtering, there were 2, 26, and 14 candidates in group 1, group 2, and group 3, respectively. The candidate antigens were further characterized using the single-cell transcriptomic data from the Malaria Cell Atlas^40–42^ that contained 12 life stages, including five sporozoite stages, three blood stages, three gametocyte stages, and one ookinete stage. The gene counts were normalized by size factors and log_2_-transformed. The proportion of cells at each stage having gene counts larger than zero, and the median and mean gene counts in the cell populations were reported. Further, the closest reference antigen to each candidate antigen based on the proximity matrix was identified. The final data set contained probability scores, clustering groups, gene products from PlasmoDB^36^ release 59 (2022-08-30), closest reference antigen and the corresponding Euclidean distance. See Supplementary Data 3 for detailed information.

### Statistical analyses

R version 4.2.1 (2022-06-23) and RStudio were used to perform statistical analyses. For comparing the scores and Euclidean distances of antigen proteins and candidate antigens from models with or without tree filtering, a pairwise two-tailed Mann–Whitney test was used. For comparisons of variable values between target proteins (known or candidate antigens) and randomly selected non-antigens, or comparisons of difference in distances across the three candidate antigen groups, a regular two-tailed Mann-Whitney test was conducted. Where appropriate, the *p*-values for multiple tests were adjusted using the Benjamini–Hochberg procedure^117^.

### Reporting summary

Further information on research design is available in the Nature Research Reporting Summary linked to this article.

### Supplementary information

Description of additional supplementary files

Supplementary Data 1

Supplementary Data 2

Supplementary Data 3

Supplementary Data 4

Supplementary Data 5

Supplementary Data 6

Reporting summary

Supplementary Information

## Data Availability

The database file, raw data of all figures and tables, and the research notebook can be found in an open-source repository (10.13016/me1l-1ahr). The interactive candidate antigen table is also available online at (https://mrp-bioinformatics.github.io/malaria_antigen_candidates/).

## Code availability

The implementation of the positive-unlabeled random forest algorithm is available as a Python package on GitHub (https://github.com/RTChou/purf).
