# Supplementary material for: Positive-unlabeled learning identifies vaccine candidate antigens in the malaria parasite Plasmodium falciparum
Source: NPJ Syst Biol Appl. 2024 Apr 27;10:44. doi: 10.1038/s41540-024-00365-1 (PMC11055854; doi:10.1038/s41540-024-00365-1)
Supplement: Supplementary file 1 — Description of additional supplementary files [file 41540_2024_365_MOESM1_ESM.docx]

**Description of Additional Supplementary Files**

File name: Supplementary Data 1

Description: Descriptions of protein variables used in machine learning

File name: Supplementary Data 2

Description: Machine learning input data set

File name: Supplementary Data 3

Description: Antigen candidate further characterizations

File name: Supplementary Data 4

Description: PURF model out-of-bag predictions

File name: Supplementary Data 5

Description: Validation results for non-tree filtered PURF model

File name: Supplementary Data 6

Description: Validation results for tree filtered PURF model
