# Supplementary material for: Positive-unlabeled learning identifies vaccine candidate antigens in the malaria parasite Plasmodium falciparum
Source: NPJ Syst Biol Appl. 2024 Apr 27;10:44. doi: 10.1038/s41540-024-00365-1 (PMC11055854; doi:10.1038/s41540-024-00365-1)
Supplement: Supplementary file 9 — Supplementary Information [file 41540_2024_365_MOESM9_ESM.pdf]

Positive-unlabeled learning identifies vaccine candidate antigens in the malaria parasite *Plasmodium falciparum*

Renee Ti Chou[1], Amed Ouattara[2], Matthew Adams[2], Andrea A. Berry[2], Shannon Takala-Harrison[2†], and Michael P. Cummings[1†]

[1]Center for Bioinformatics and Computational Biology, University of Maryland, College Park, College Park, MD, USA
[2]Center for Vaccine Development and Global Health, University of Maryland School of Medicine, Baltimore, MD, USA
[†]Corresponding authors. e-mail: mcummin1@umd.edu; stakala@som.umaryland.edu

Contents

1 Supplementary Notes **4**

2 Supplementary Figures **5**

3 Supplementary Tables **16**

List of Figures

Supplementary Fig. 1 5
Supplementary Fig. 2 6
Supplementary Fig. 3 7
Supplementary Fig. 4 8
Supplementary Fig. 5 9
Supplementary Fig. 6 10
Supplementary Fig. 7 11
Supplementary Fig. 8 12
Supplementary Fig. 9 13
Supplementary Fig. 10 14
Supplementary Fig. 11 15

List of Tables

Supplementary Table 1 16
Supplementary Table 2 17
Supplementary Table 3 18

1 Supplementary Notes

All supplementary materials including the database file, raw data, and the research notebook are available in the Digital Repository at the University of Maryland (DRUM), http://hdl.handle.net/1903/29775. The research notebook contains instructions on installing the PURF package, retrieving protein variables and assembling machine leaning input from the database, as well as code for experimental analysis and plotting. To open the research notebook, go to the subfolder `main_notebook`, and click on the HTML file `index.html` to open it in a web browser. A PDF version of the notebook `main_notebook.pdf` is also available. To run the code in the research notebook, open R Markdown (.Rmd) files in RStudio. Data generated from the notebook are stored in the subfolders `other_data` (structured data), `pickle_data` (Python objects), and `rdata` (R objects).

2 Supplementary Figures

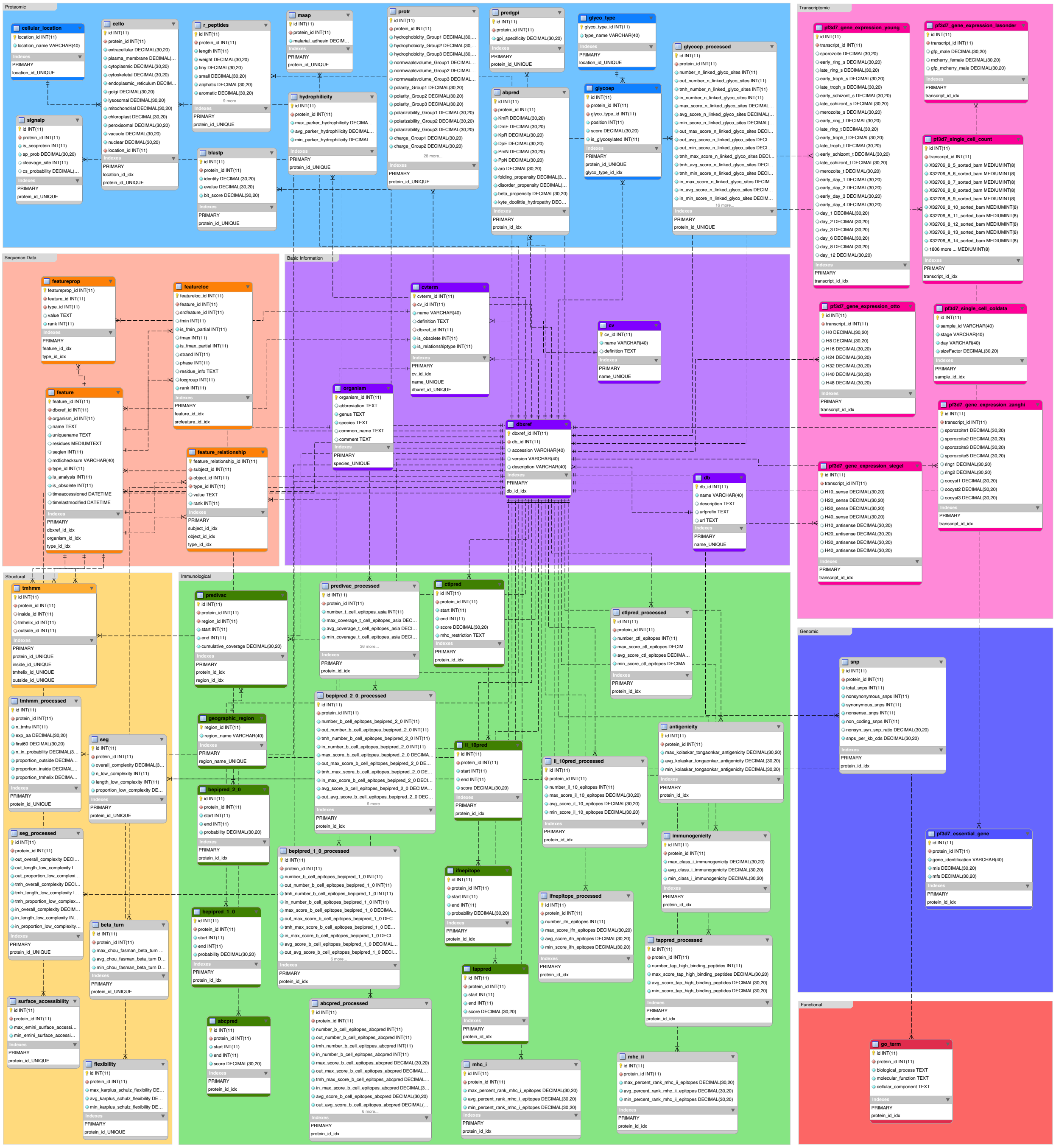

Supplementary Fig. 1 | Database schema of *P. falciparum* reverse vaccinology data. Data tables were grouped based on data properties and shown in different colors (light blue: proteomic; orange: sequence data; purple: basic information; yellow: structural; green: immunological; pink: transcriptomic; dark blue: genomic). Lines indicate relationship between data tables. Each table shows the variables stored and their corresponding data types in the database context. Data tables used to assemble the input data set used in the study are shown in grey.

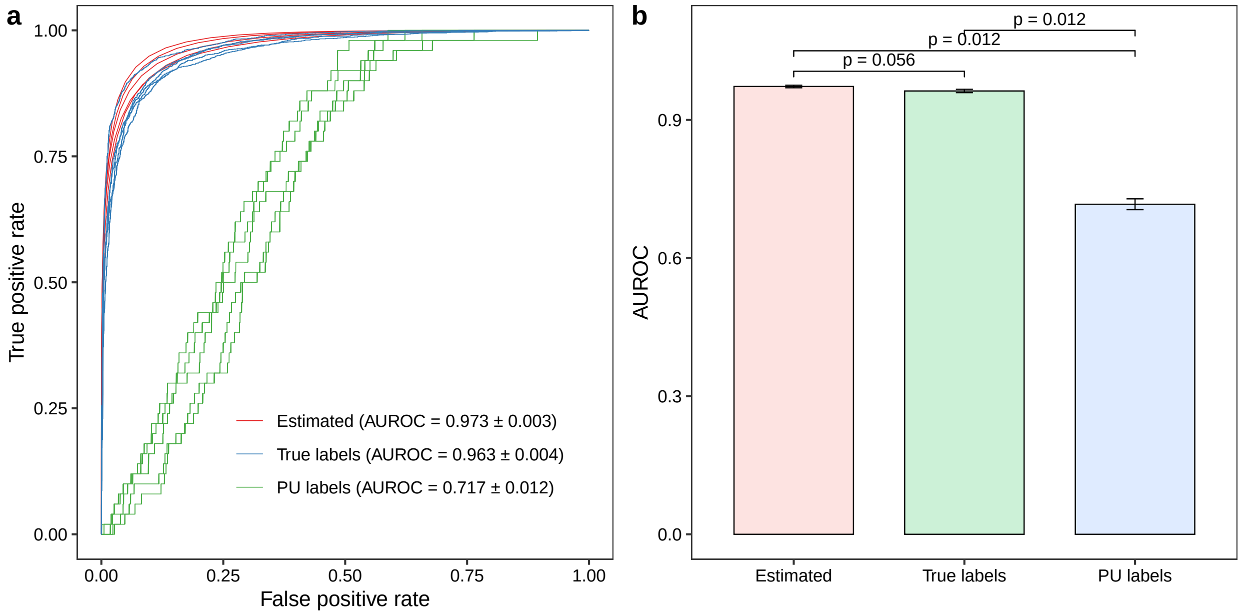

Supplementary Fig. 2 | Evaluation of model performance on simulated data set. a, Lines represent receiver operating characteristic (ROC) curves estimated from the prediction score distribution (red), as well as computed regarding true labels and positive-unlabeled (PU) labels (blue and light green, respectively). The areas under the receiver operating characteristic curve (AUROC; mean ± SEM; $n = 5$) are noted in the parentheses in the legend. **b,** Barplots showing the AUROC of ROC curves in **a**. Error bars indicate standard errors. Mann–Whitney test (two-sided) was performed, and the adjusted p values are shown on the top of the bars.

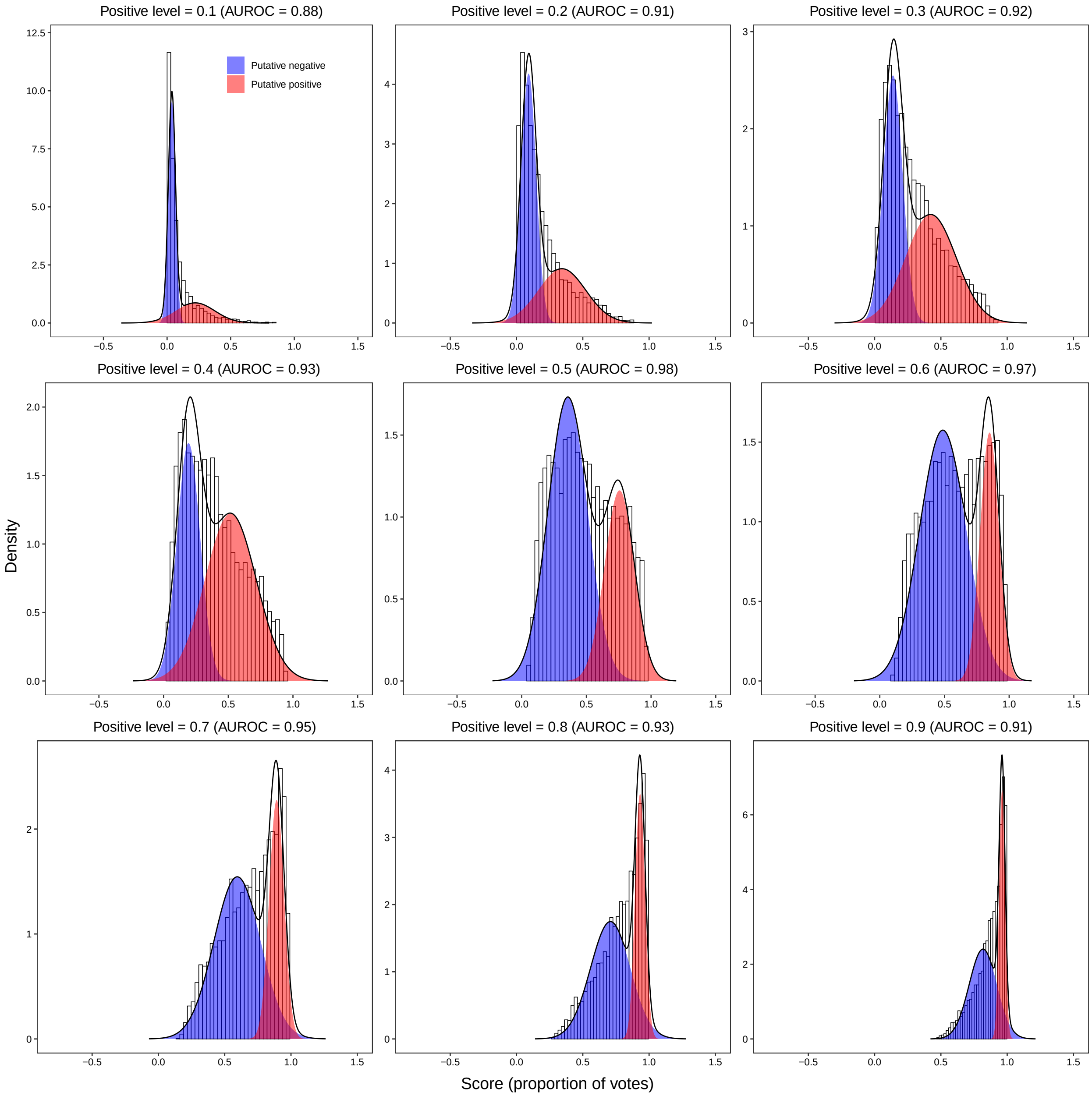

Supplementary Fig. 3 | Hyper-parameter tuning before variable space weighting. Subplots show prediction score distributions of the unlabeled proteins. The putative positive (red) and negative (blue) groups were computed using a two-component Gaussian mixture model. Receiver operating characteristic (ROC) curves were calculated based on the estimated distribution groups, and the areas under the receiver operating characteristic curves (AUROC) are indicated in the parentheses in the subplot titles.

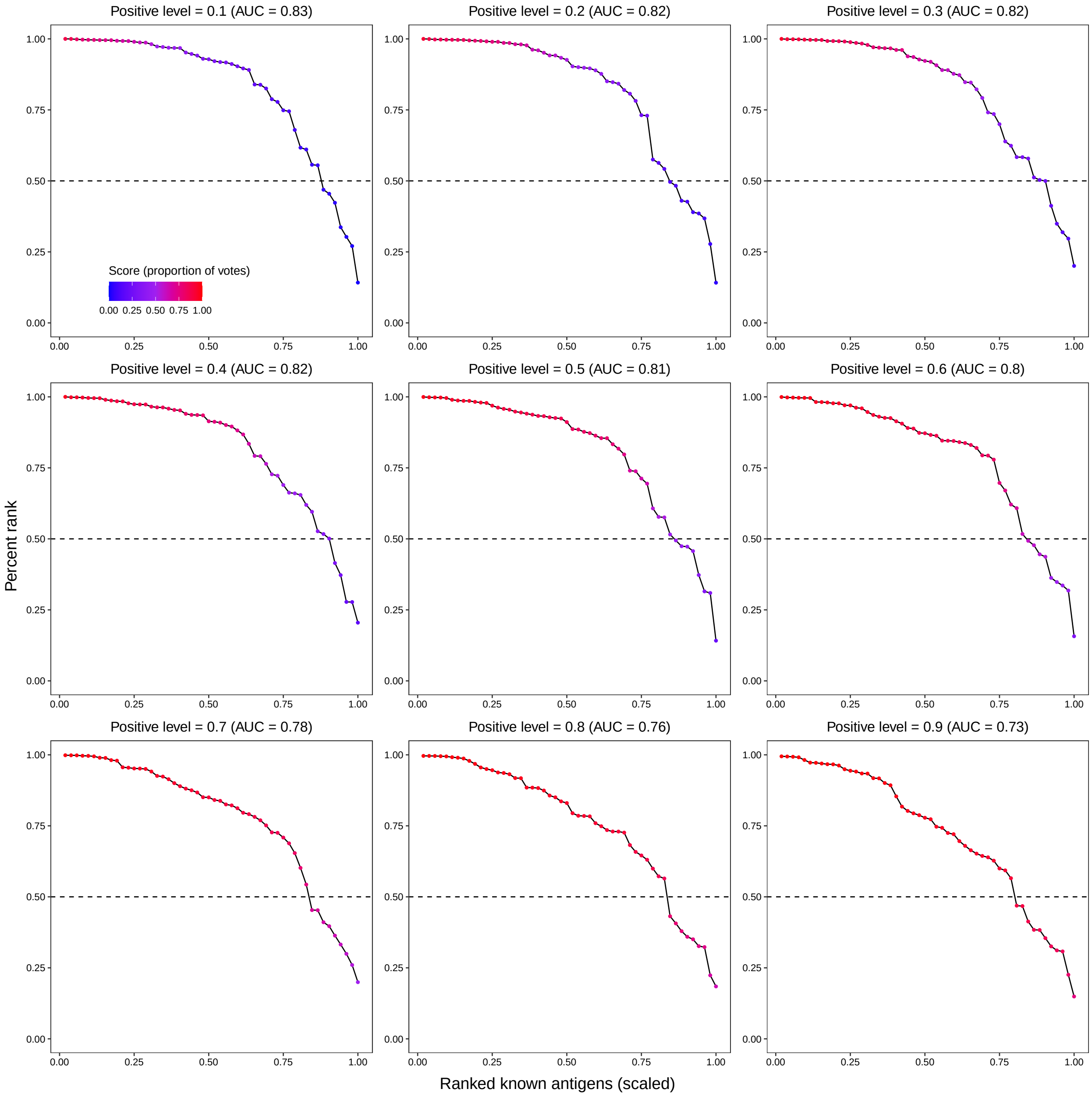

Supplementary Fig. 4 | Evaluation of known antigen predictions before variable space weighting. known antigens (n = 52) and unlabeled proteins were ranked based on probability scores from ensembles with different positive levels (0.1–0.9; shown as subplots). The x-axes show scaled ranks of known antigens, and the y-axes indicate precentile rank of known antigens among all *P. falciparum* proteins. Gradient colors represent prediction scores. Dashed lines indicated percentile ranks of 0.5. The areas under the ranking curves (AUC) are shown in the parenthesis in the subplot titles.

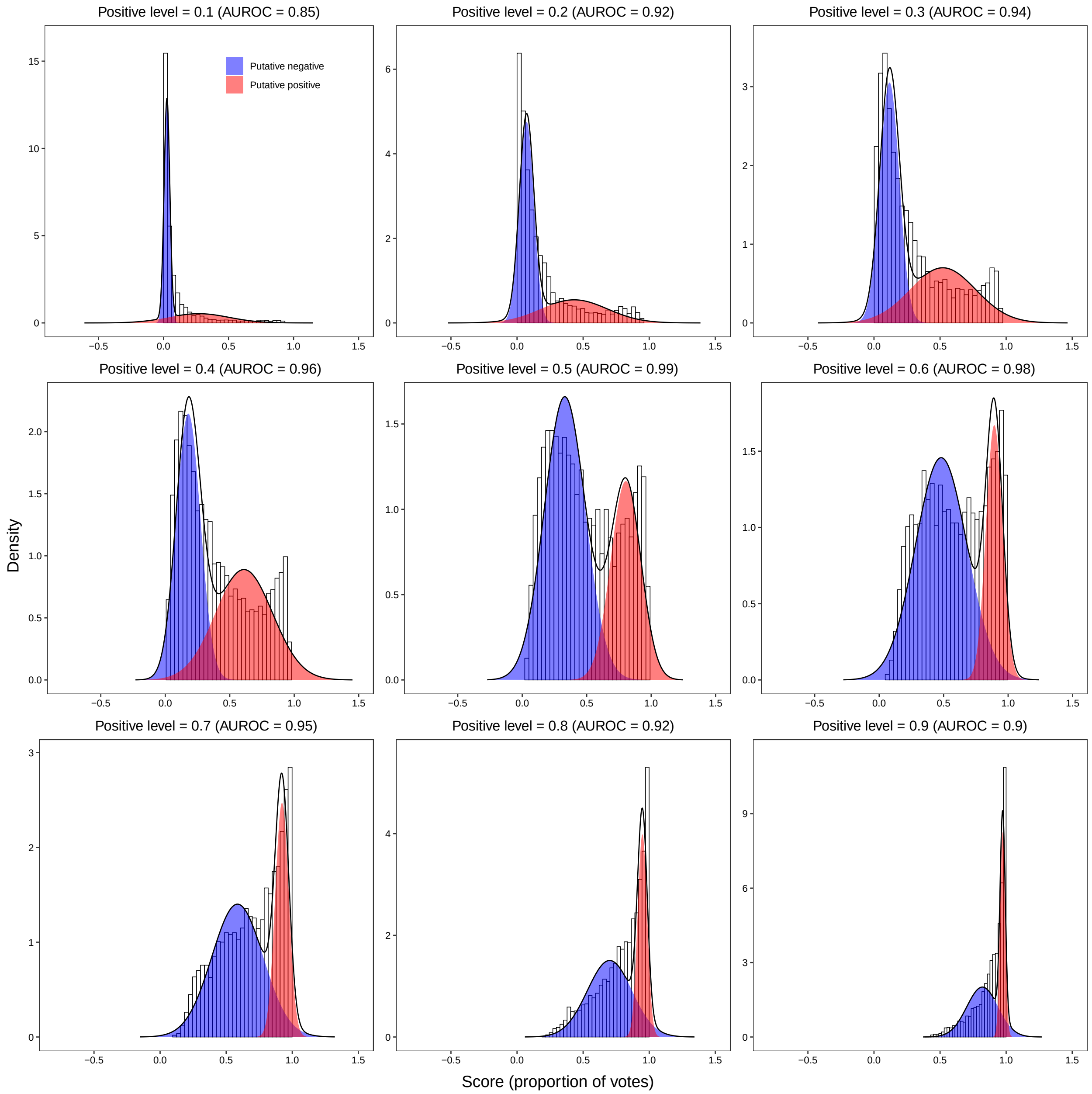

Supplementary Fig. 5 | Hyper-parameter tuning after variable space weighting. Probability score distributions of unlabeled proteins predicted by ensembles with different positive levels are shown in subplots. Score distributions were fitted using a two-component Gaussian mixture model to estimate the putative positive (red) and negative (blue) groups. Receiver operating characteristic curves (ROC) were calculated from the estimated distributions. The areas under the receiver operating characteristic curves (AUROC) are noted in the parentheses following the subplot titles.

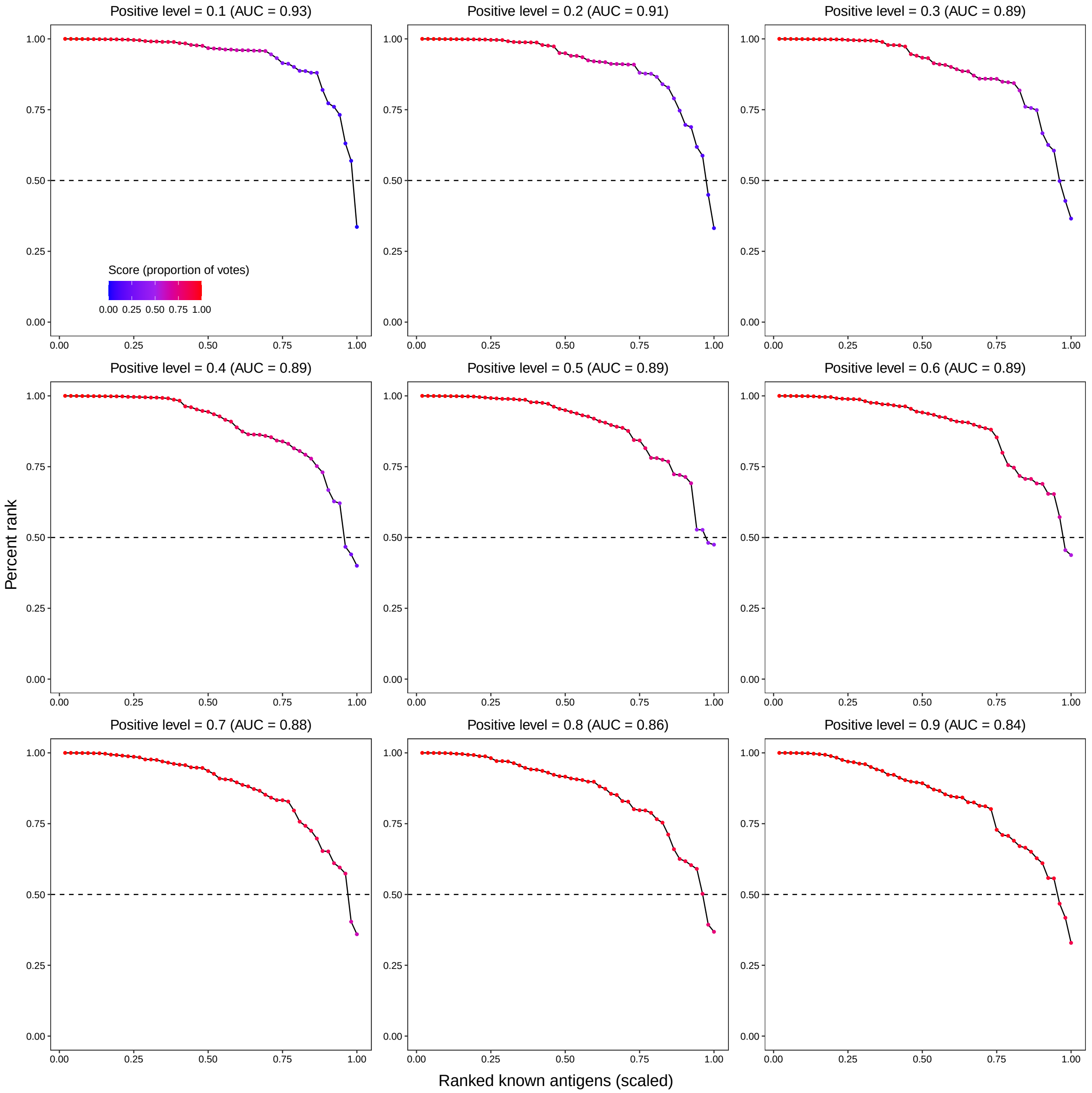

Supplementary Fig. 6 | Evaluation of known antigen predictions after variable space weighting. Dots in the subplots represent known antigens ($n = 52$). The x-axes show scaled ranks of known antigens only. The y-axes represent percentile ranks of known antigens among all *P. falciparum* proteins. Probability scores are noted by gradient colors. Dashed lines show 0.5 percent ranks, and the areas under the curves (AUC) are shown in the subplot title parentheses.

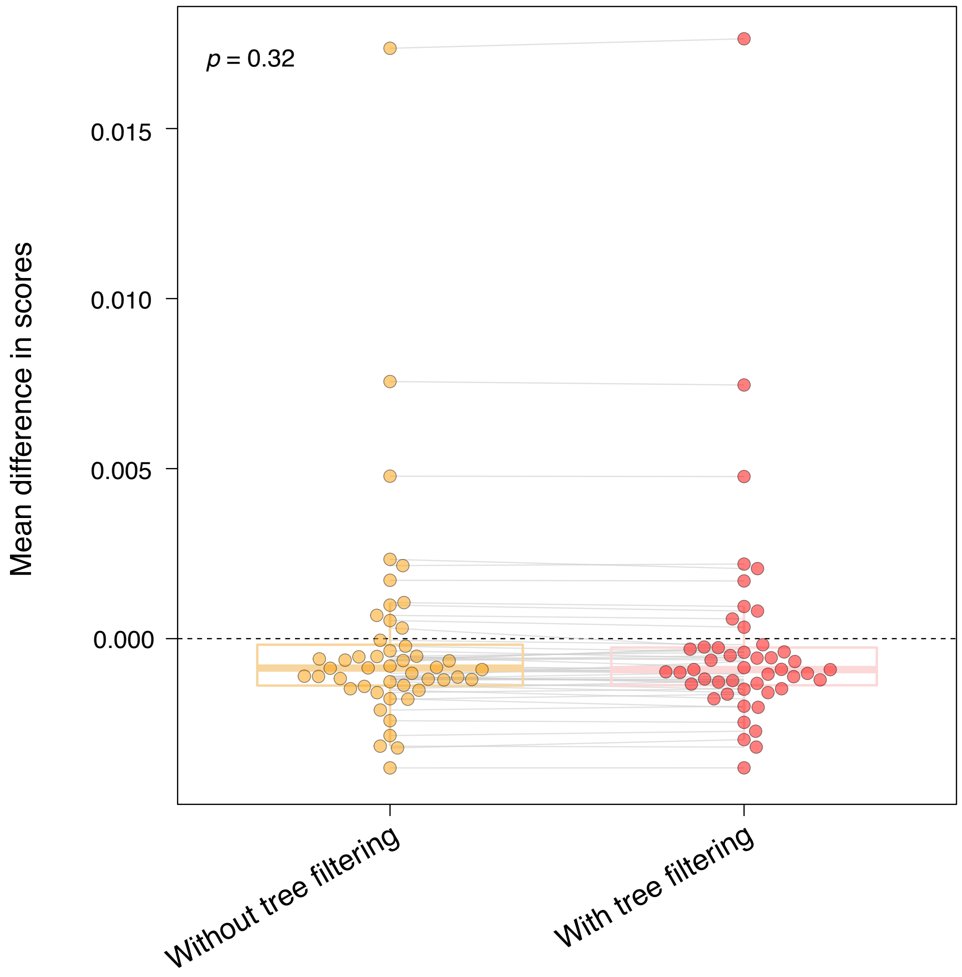

Supplementary Fig. 7 | Comparison of mean differences in probability scores after known antigen label removal. Labels of known antigens were removed iteratively, and the mean differences in scores for the remaining known antigens were calculated. The validation procedure was performed for ensembles with (red points, $n = 48$) and without (yellow points, $n = 48$) tree filtering. The box plots indicate medians with first and third quartiles. The lower and upper whiskers show 1.5 times the interquartile range extended from the first and third quartiles, respectively. The grey lines connect the same label removal iteration in both distributions. The dashed line shows a zero-mean difference in scores. The p-value was calculated using a pairwise two-sided Mann–Whitney test.

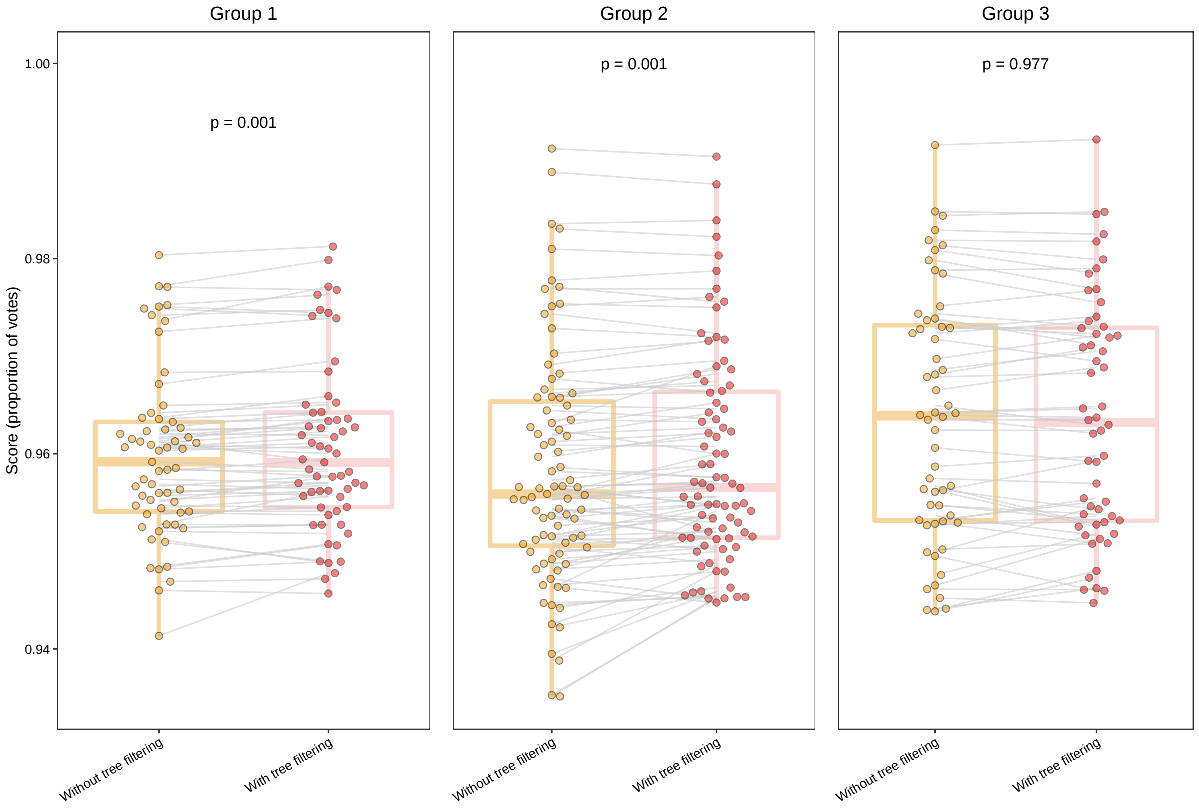

Supplementary Fig. 8 | Probability scores of candidate antigen groups. Comparison of scores predicted by non-tree-filtered (yellow points) and tree-filtered (red points) models for the three candidate groups (samples sizes: 61, 83, and 56). Points represent candidate antigens. Boxplots show the medians with the first and third quartiles. The lower and upper whiskers indicate 1.5-times the interquartile range extended from the first and third quartiles, respectively. Grey lines connect pairs of the same candidate antigens in the group, and adjusted p-values from two-sided pairwise Mann–Whitney tests are noted.

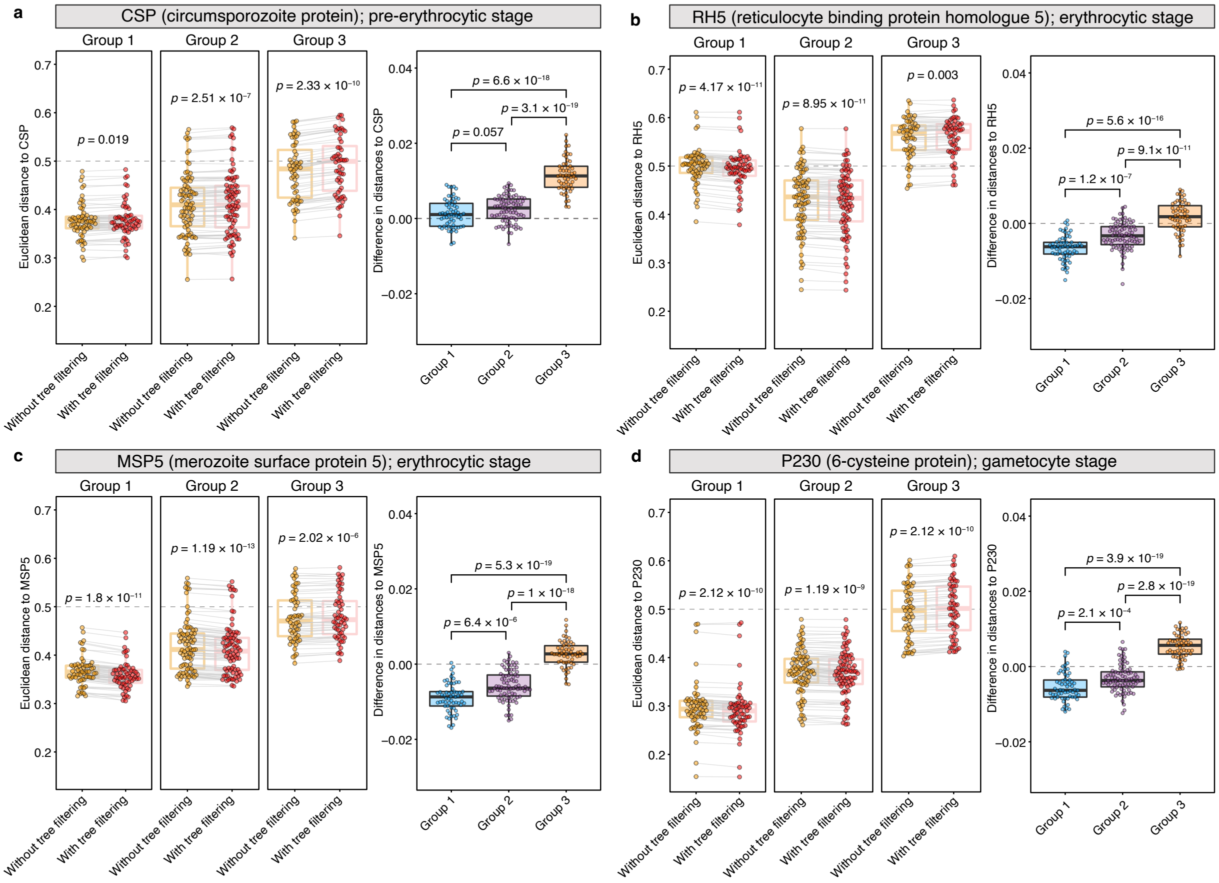

Supplementary Fig. 9 | Statistical comparisons of distances between candidate and reference antigens. Euclidean distances (ranging from 0–1) were calculated from the proximity matrix from the tree-based models. The summaries of distances of candidate antigens to the reference antigens CSP, RH5 MSP5, and P230 are shown in **a**, **b**, **c**, and **d**, respectively. For each plot, the left three panels show comparisons (two-sided pairwise Mann–Whitney test; p-values adjusted using the Benjamini–Hochberg procedure) of distances computed from the non-tree-filtered (yellow points) and tree-filtered (red points) models. The rightmost panel shows the comparisons (two-sided Mann–Whitney test with p-values adjusted) of distance differences between the three candidate antigen groups before and after tree filtering. Points represent candidate antigens. The corresponding p-values are noted above the compared groups. Dashed lines in the left and right panels indicate 0.5 distance and 0 difference in distances, respectively.

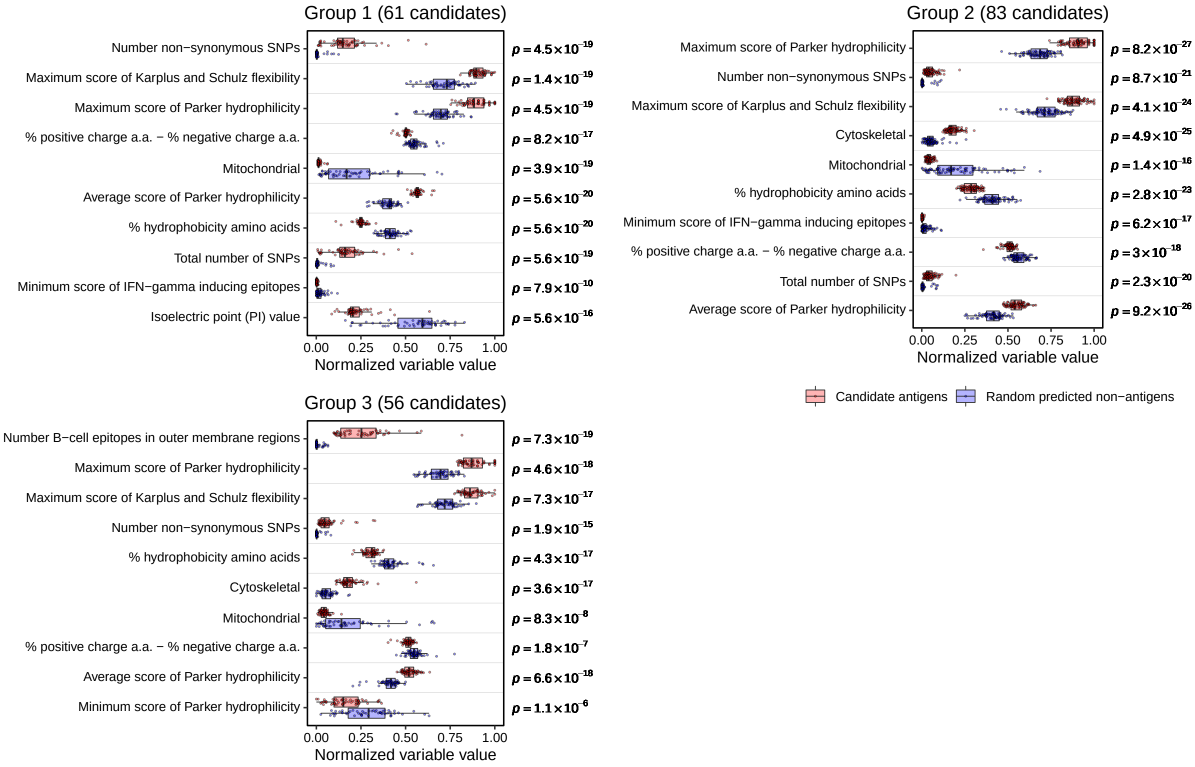

Supplementary Fig. 10 | Statistical comparisons of variable values of top important variables between the candidate antigen groups and randomly selected non-antigens. Top ten important variables analyzed using permutation-based variable importance based on the candidates in each group are shown (see also **Supplementary Tables 1–3**). For each candidate antigen group, the variable values were compared with a same number of randomly selected non-antigens predicted (probability score < 0.5) using a two-sided Mann–Whitney test adjusted by the Benjamini-Hochberg procedure. The x-axes show normalized variable values based on the whole data set. Red dots and blue dots indicate candidates and predicted non-antigens, respectively. Boxplots display the first quartile, median, and third quartile values. The left and right whiskers indicate the 1.5 interquartile range extended from the first and third quartiles, respectively.

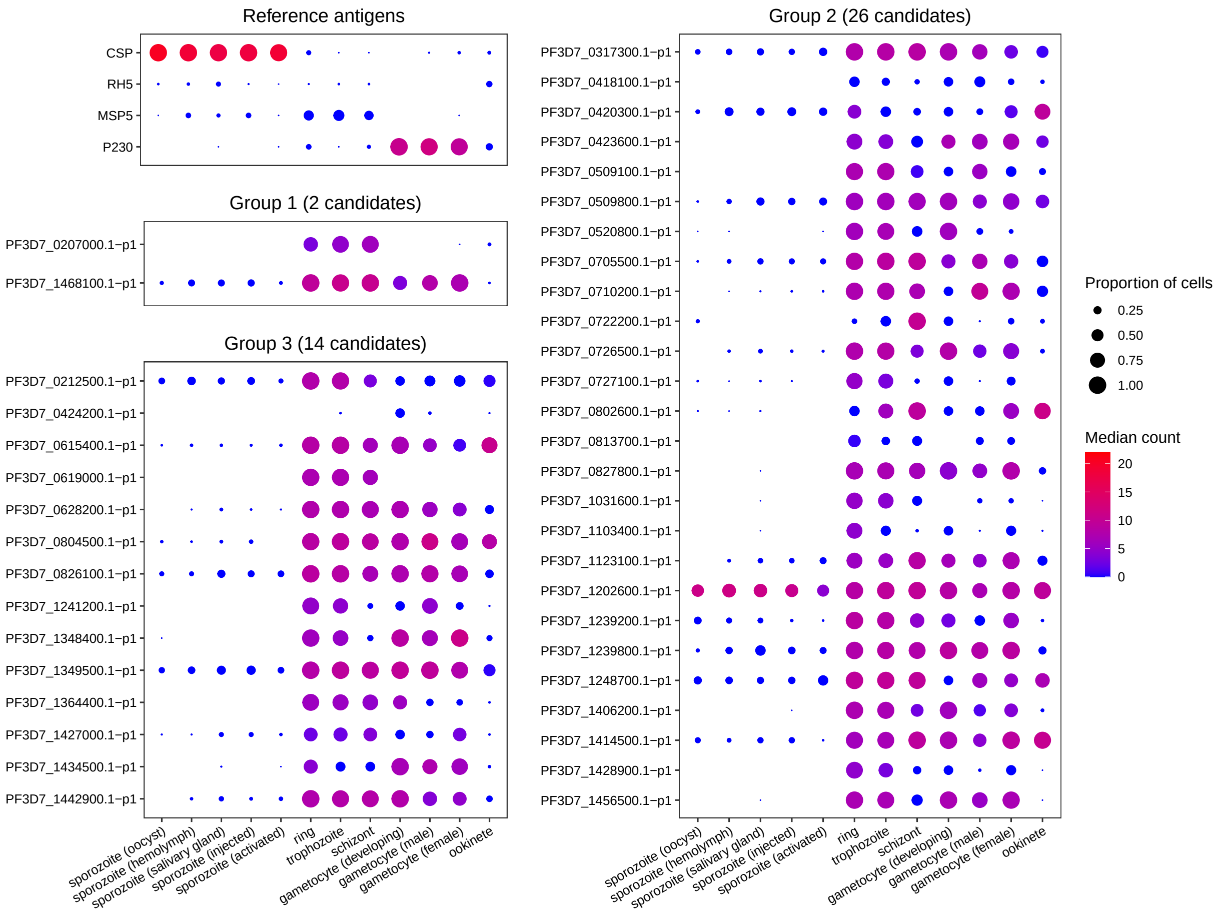

Supplementary Fig. 11 | Candidate antigen characterization across various *P. falciparum* life stages. Normalized gene counts of cells in each stage for the four reference antigens and the filtered candidate antigens with single-cell transcriptomic data from the Malaria Cell Atlas [1–3] are shown. Dot size represents proportion of cells having gene count larger than zero. Gradient colors indicate median count of the cell population in each life stage. The numbers of antigens in each candidate antigen group are noted in the parentheses in the subplot titles.

3 Supplementary Tables

Supplementary Table 1 | Top important variables (upper part) and variable categories (lower part) in group 1 candidate antigens. Ranks in groups 2 and 3 individual variable and variable category importance are also shown (MDA: Mean Decrease Accuracy)

**#**	**Variable**	**MDA**	**Group 2 rank**	**Group 3 rank**	**Group**
1	Number non-synonymous SNPs	46.94	2	4	Genomic
2	Maximum score of Karplus and Schulz flexibility	46.52	3	3	Structural
3	Maximum score of Parker hydrophilicity	39.88	1	2	Proteomic
4	% positive charge a.a. − % negative charge a.a.	35.75	8	8	Proteomic
5	Mitochondrial	35.42	5	7	Proteomic
6	Average score of Parker hydrophilicity	35.29	10	9	Proteomic
7	% hydrophobicity amino acids	32.59	6	5	Proteomic
8	Total number of SNPs	32.34	9	12	Genomic
9	Minimum score of IFN-γ inducing epitopes	30.07	7	11	Immunological
10	Isoelectric point (PI) value	29.31	21	15	Proteomic
**#**	**Group variable**	**MDA**	**Group 1 rank**	**Group 2 rank**	
1	Proteomic group variables	174.69	1	1	
2	Immunological group variables	106.54	2	2	
3	Structural group variables	64.64	3	3	
4	Genomic group variables	58.52	4	4	

Supplementary Table 2 | Top important variables (upper part) and variable categories (lower part) in group 2 candidate antigens. Ranks in groups 1 and 3 variable and variable category importance are also shown (MDA: Mean Decrease Accuracy)

#	Variable	MDA	Group 1 rank	Group 3 rank	Group
1	Maximum score of Parker hydrophilicity	52.05	3	2	Proteomic
2	Number non-synonymous SNPs	51.85	1	4	Genomic
3	Maximum score of Karplus and Schulz flexibility	51.45	2	3	Structural
4	Cytoskeletal	43.65	11	6	Proteomic
5	Mitochondrial	41.69	5	7	Proteomic
6	% hydrophobicity amino acids	40.30	7	5	Proteomic
7	Minimum score of IFN-γ inducing epitopes	38.95	9	11	Immunological
8	% positive charge a.a. $-$ % negative charge a.a.	38.20	4	8	Proteomic
9	Total number of SNPs	38.01	8	12	Genomic
10	Average score of Parker hydrophilicity	35.37	6	9	Proteomic
**#**	**Group variable**	**MDA**	**Group 1 rank**	**Group 2 rank**	
1	Proteomic group variables	195.21	1	1	
2	Immunological group variables	124.44	2	2	
3	Structural group variables	76.17	3	3	
4	Genomic group variables	73.78	4	4	

Supplementary Table 3 | Top important variables (upper part) and variable categories (lower part) in group 3 candidate antigens. Ranks in groups 1 and 2 variable and variable category importance are also shown (MDA: Mean Decrease Accuracy)

#	Variable	MDA	Group 1 rank	Group 2 rank	Group
1	Number B-cell epitopes in outer membrane regions	59.60	272	272	Immunological
2	Maximum score of Parker hydrophilicity	50.61	3	1	Proteomic
3	Maximum score of Karplus and Schulz flexibility	47.96	2	3	Structural
4	Number non-synonymous SNPs	43.77	1	2	Genomic
5	% hydrophobicity amino acids	41.91	7	6	Proteomic
6	Cytoskeletal	41.48	11	4	Proteomic
7	Mitochondrial	38.95	5	5	Proteomic
8	% positive charge a.a. − % negative charge a.a.	37.23	4	8	Proteomic
9	Average score of Parker hydrophilicity	36.71	6	10	Proteomic
10	Minimum score of Parker hydrophilicity	36.46	257	11	Proteomic
**#**	**Group variable**	**MDA**	**Group 1 rank**	**Group 2 rank**	
1	Proteomic group variables	177.86	1	1	
2	Immunological group variables	157.07	2	2	
3	Structural group variables	88.26	3	3	
4	Genomic group variables	62.82	4	4	

References

[1] V. M. Howick, A. J. C. Russell, T. Andrews, H. Heaton, A. J. Reid, K. Natarajan, H. Butungi, T. Metcalf, L. H. Verzier, J. C. Rayner, M. Berriman, J. K. Herren, O. Billker, M. Hemberg, A. M. Talman, and M. K. N. Lawniczak. The Malaria Cell Atlas: single parasite transcriptomes across the complete *Plasmodium* life cycle. *Science*, 365(6455), 08 2019. PMCID: PMC7056351.

[2] E. Real, V. M. Howick, F. A. Dahalan, K. Witmer, J. Cudini, C. Andradi-Brown, J. Blight, M. S. Davidson, S. K. Dogga, A. J. Reid, J. Baum, and M. K. N. Lawniczak. A single-cell atlas of *Plasmodium falciparum* transmission through the mosquito. *Nat Commun*, 12(1):3196, May 2021. PMCID: PMC8159942.

[3] A. J. Reid, A. M. Talman, H. M. Bennett, A. R. Gomes, M. J. Sanders, C. J. R. Illingworth, O. Billker, M. Berriman, and M. K. Lawniczak. Single-cell RNA-seq reveals hidden transcriptional variation in malaria parasites. *Elife*, 7, Mar 2018. PMCID: PMC5871331.
